# Cardiovascular magnetic resonance using T1-mapping, T2-weighted and late gadolinium enhancement imaging provides a high diagnostic yield in patients presenting with acute chest pain, positive troponin and non-obstructive coronary arteries

**DOI:** 10.1186/1532-429X-16-S1-P215

**Published:** 2014-01-16

**Authors:** Vanessa M Ferreira, Erica Dall'Armellina, Stefan K Piechnik, Theodoros D Karamitsos, Jane M Francis, Robin Choudhury, Keith Channon, Rajesh Kharbanda, Colin Forfar, Oliver Ormerod, Bernard D Prendergast, Attila Kardos, Jim Newton, Matthias G Friedrich, Matthew D Robson, Stefan Neubauer

**Affiliations:** 1 Division of Cardiovascular Medicine, Radcliffe Department of Medicine, University of Oxford, Oxford, UK; 2 Department of Cardiology, Milton Keynes NHS Hospital Foundation trust, Milton Keynes, UK; 3 Department of Cardiology, Université de Montréal, Montréal, Quebec, Canada; 4 Stephenson Cardiovascular MR Centre, Libin Cardiovascular Institute of Alberta, University of Calgary, Calgary, Alberta, Canada

## Background

Patients presenting with chest pain, raised troponin but non-obstructive coronary arteries pose a clinical challenge in diagnosis, prognosis and management. We hypothesized that early, multiparametric cardiovascular magnetic resonance (CMR) imaging can localize areas of injury and provide a diagnosis these cases.

## Methods

**Figure 1:**
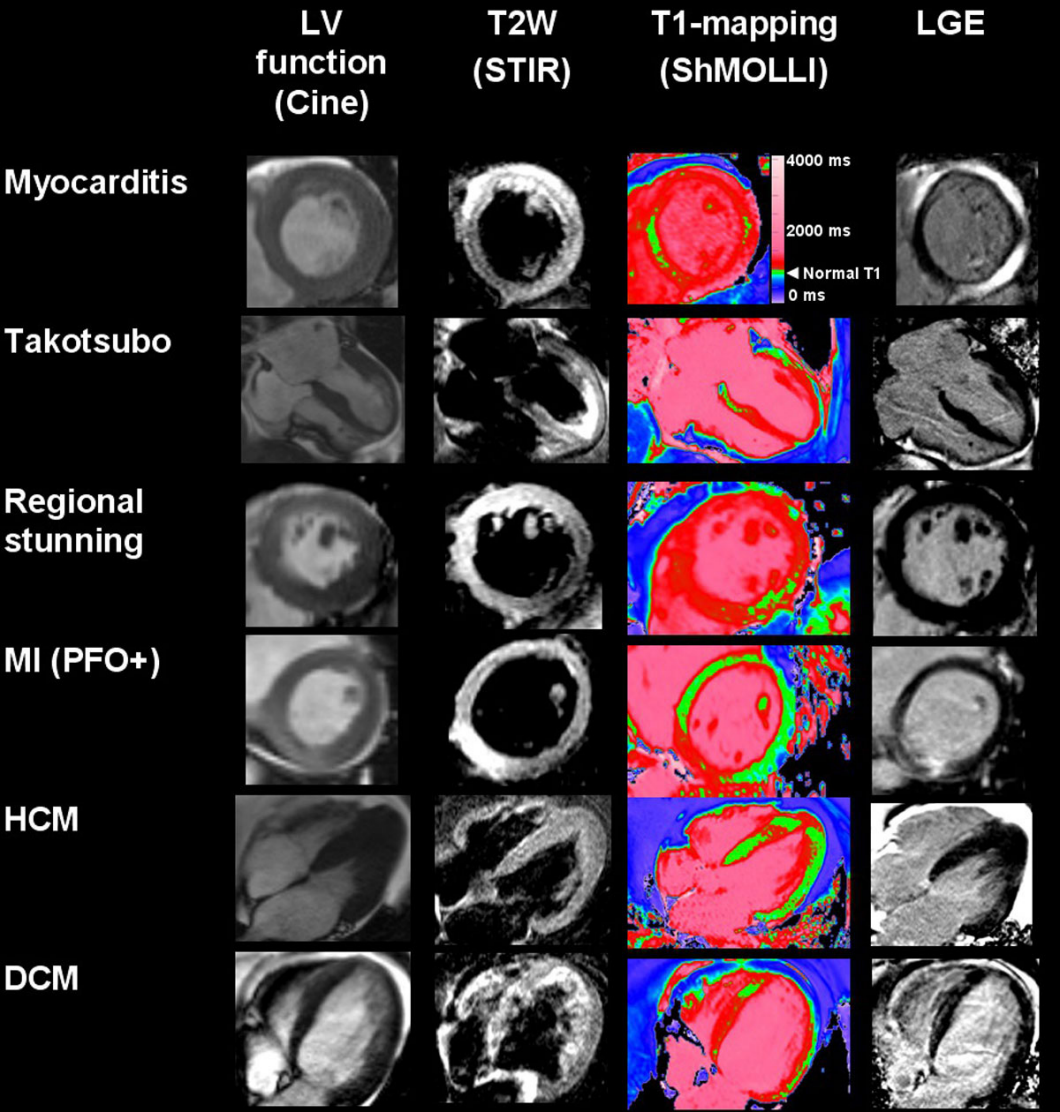
**Early CMR using multiparametric tissue characterization including cine, T2-weighted (T2W), T1-mapping and late gadolinium enhancement imaging to assess patients presenting with acute chest pain, positive troponins and non-obstructive coronary arteries**. On T1-maps, green areas within the LV denote normal myocardium; red areas have a T1> 990 ms, consistent with acute injury.

We prospectively studied 120 patients (mean age 50 ± 17 yrs; 50% female) presenting with chest pain, positive troponin I (normal <0.04, median 3.99, range 0.07 to >60 μg/L) and non-obstructive coronaries. Early CMR at 1.5T (median 3 days, IQR 1-6 days) included cine, dark-blood T2-weighted (STIR), native T1-mapping (ShMOLLI) and late gadolinium enhancement (LGE) imaging (Figure 1). Findings were compared to 50 controls. Image analysis included: the detection of edema comparing T2 signal intensity of myocardium to skeletal muscle (>2.0) or remote myocardium (>2 SD); myocardial T1 times (areas of injury with an area of ≥40 mm2 with T1 >990 ms); and presence of LGE.

## Results

**Figure 2:**
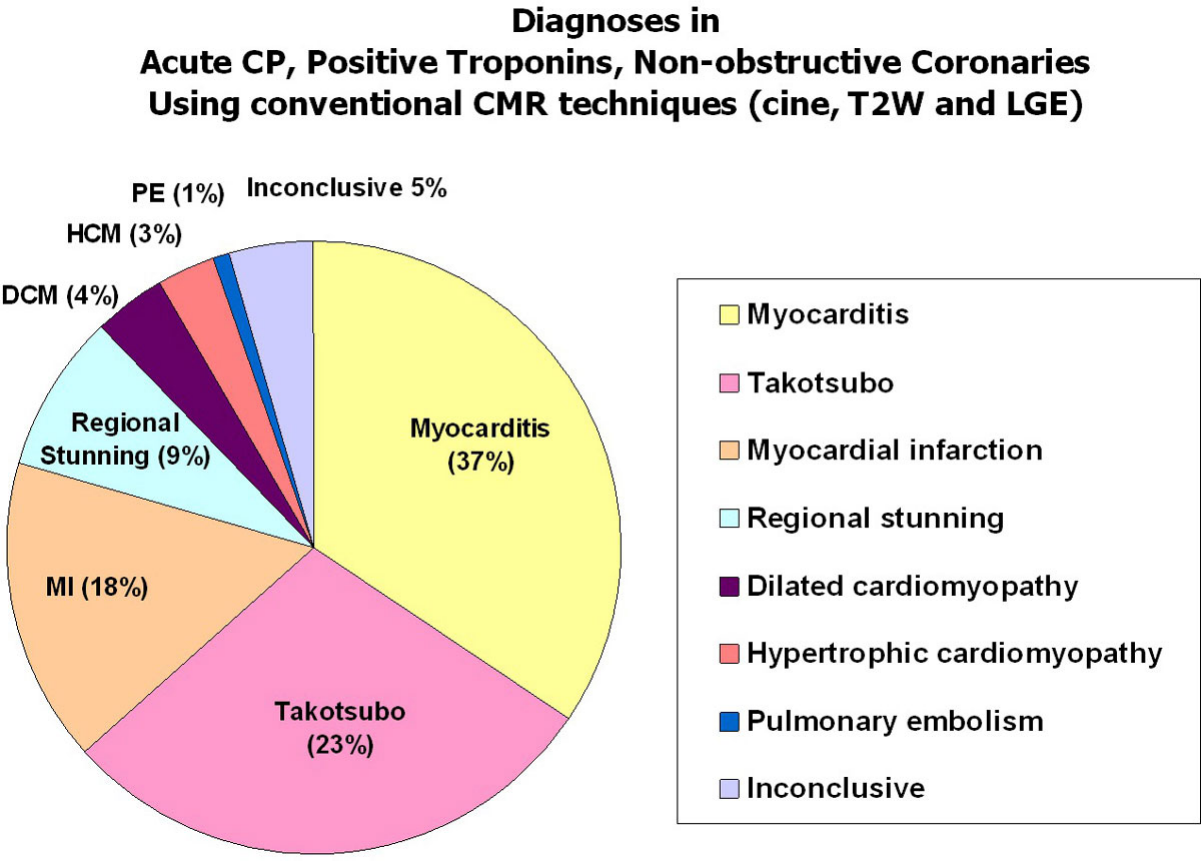
**CMR, when performed early, provides a high diagnostic yield in patients presenting with acute chest pain, positive troponins and non-obstructive coronaries even using just conventional techniques (cine, T2-weighted and late gadolinium enhancement imaging)**.

Using only conventional CMR techniques (cine, T2W and LGE; Figure 2), there was a high diagnostic yield of 95%. Edema was detected in 79% and LGE in 61% of patients. Based on CMR findings including the type, pattern and regional distribution of injury, the commonest diagnosis was myocarditis (37%), followed by Takotsubo cardiomyopathy (23%), myocardial infarction (18%), acute regional stunning (9%; wall motion abnormality with edema but no LGE), dilated cardiomyopathy (4%), hypertrophic cardiomyopathy (3%), and missed pulmonary embolism (1%). In 11/21 (52%) of patients with MI, a patent foramen ovale (PFO) was demonstrated on echocardiography with agitated saline contrast, suggesting these patients may have suffered a paradoxical coronary embolism. The remaining 5.0% (n = 6) of patients had no findings on T2W and LGE imaging. However, native T1-mapping identified areas of injury (T1 >990 ms) in 4 out of the remaining 6 patients, improving the detection rate to 98%.

## Conclusions

Using conventional T2W and LGE techniques, early CMR has a high diagnostic yield (95%) in patients presenting with troponin-positive chest pain but non-obstructive coronary arteries. Native T1-mapping detected additional areas of abnormality when conventional CMR was "normal", improving the detection rate to 98%. Early multiparametric CMR is able to localize areas of affected myocardium and may aid in the further management or diagnostic workup in this patient cohort.

## Funding

This study is funded by the Oxford National Institute for Health Research Biomedical Research Centre Programme. VMF received funding from the Alberta Innovates Health Solutions (AIHS) Clinical Fellowship and the University of Oxford Clarendon Fund Scholarship. RC is a Wellcome Trust Senior Research Fellow in Clinical Science. SN and RC acknowledge support from the British Heart Foundation Centre of Research Excellence, Oxford.

